# Care intervention on psychological outcomes among patients admitted to intensive care unit: an umbrella review of systematic reviews and meta-analyses

**DOI:** 10.1186/s13643-023-02372-5

**Published:** 2023-12-14

**Authors:** Yafang Zheng, Lijuan Zhang, Shihong Ma, Bian Wu, Peipei Chen, Yan Xu, Wenting Tan, Hanzhan Li, Qiaomei Wu, Jingxia Zheng

**Affiliations:** 1 The Second Affiliated Hospital of Guangzhou University of Chinese Medicine, Guangzhou, People’s Republic of China; 2 Guangdong Provincial Hospital of Traditional Chinese Medicine, No. 111 Dade Road, Guangzhou, 510120 People’s Republic of China

**Keywords:** Intensive care unit, Care intervention, Psychological health, Umbrella review

## Abstract

**Background:**

Numerous studies have explored care interventions to improve the psychological outcome of intensive care unit (ICU) patients, but inconclusive evidence makes it difficult for decision-makers, managers, and clinicians to get familiar with all available literature and find appropriate interventions. This umbrella review aimed to analyze the relationship between care intervention and psychological outcomes of ICU patients based on existing systematic reviews.

**Methods:**

An umbrella review of evidence across systematic reviews and meta-analyses published between 1987 and 2023 was undertaken. We systematically searched reviews that examined the association between care intervention and the improvement of adverse psychological outcomes in ICU patients using PubMed, EMBASE, Web of Science, Cochrane Library, and manual reference screening. The measurement tool (AMSTAR 2) was applied to evaluate the methodological quality of included studies. The excess significance bias, between-study heterogeneity expressed by *I*^2^, small-study effect, and evidence class were estimated.

**Results:**

A total of 5110 articles were initially identified from the search databases and nine of them were included in the analysis. By applying standardized criteria, only weak evidence was observed in 13 associations, even though most included reviews were of moderate to high methodological quality. These associations pertained to eight interventions (music therapy, early rehabilitation, post-ICU follow-up, ICU diary, information intervention, preoperative education, communication and psychological support, surrogate decision-making) and five psychological outcomes (post-intensive care syndrome, transfer anxiety, post-traumatic stress disorder, anxiety, and depression). Weak or null association was shown among the rest of the associations (e.g., weak association between music therapy and maternal anxiety or stress level).

**Conclusions:**

The evidence of these eight supporting interventions to improve the adverse psychological outcomes of ICU patients and caregivers was weak. Data from more and better-designed studies with larger sample sizes are needed to establish robust evidence.

**Supplementary Information:**

The online version contains supplementary material available at 10.1186/s13643-023-02372-5.

## Background

Advanced technologies, instruments, and education systems used in intensive care unit (ICU) have significantly reduced the mortality of critically ill patients [1]. However, the physical and mental abilities of the surviving critically ill patients to resume normal life are impaired to a certain extent [2]. They will suffer from symptoms that affect their physical, mental, and cognitive health for a long time [3].

Several mental disorders including post-intensive care syndrome (PICS) [4], transfer anxiety [5], post-traumatic stress disorder (PTSD) [6], anxiety [7], and depression [8] are commonly found among critically ill survivors. PICS is a new syndrome that is characterized by new or deteriorated physical, cognitive, or mental health impairment after critical illness, and it could persist after acute care hospitalization [4, 9]. “Transfer anxiety” refers to the psychological and physical problems encountered by patients and their families when they are transferred from the intensive care environment to the general ward environment [5, 10]. These persistent physical, cognitive, and mental disorders experienced by ICU survivors might prevent them from returning home after being discharged from the hospital. This means that it is difficult for them to resume normal daily life when they return home. It may also further cause psychological problems such as anxiety and depression in caregivers, which will subsequently affect their ability to recover from severe diseases.

A substantial amount of literature exists on care interventions to improve psychological outcomes among patients admitted to ICU [11, 12]. Each review evaluates its own specific interventions, making it difficult for policymakers, managers, and clinicians to familiarize themselves with all available literature and to determine which interventions should be applied. Therefore, a comprehensive review of the literature is needed to identify and evaluate evidence and then select effective care interventions to improve the psychological status of patients in the ICU, as well as provide more effective suggestions to decision-makers, managers, and clinicians to improve ICU survivors’ health quality.

To achieve this, an “umbrella review” was designed to describe this approach by synthesizing the evidence of published system reviews and selecting reviews based on pre-determined criteria without an in-depth study of the quality of individual major studies included in the original system review [13]; thus, it is defined as “an overview of existing system review” [14]. A systematic review is conducted by systematically searching, evaluating, and synthesizing evidence in accordance with specific guidelines [15]. Therefore, this umbrella review aims to help managers and clinicians find solutions to problems in an evidence-based manner by summarizing the evidence from the systematic reviews and meta-analyses and improving the psychological outcomes of ICU patients.

## Methods

We performed this umbrella review systematically to collect and evaluate information from systematic reviews and meta-analyses focusing on care intervention on psychological outcomes among patients admitted to ICU. The umbrella review was carried out under the guidelines for Preferred Reporting Items for Systematic Reviews and Meta-Analyses (PRISMA). This study was not prospectively registered at the PROSPERO due to unawareness at the beginning of the study.

### Search strategy

Two reviewers independently performed a comprehensive search in four electronic databases: PubMed (from 1996 to present), EMBASE (from 1910 to present), Web of Science (from 1956 to present), and the Cochrane Library (from 1995 to present) by using the comprehensive search strategies (Table S1) from inception to January 3, 2023. We limited the search to humans and the English language. Each literature was first reviewed for title and abstract, followed by full-text retrieval of potentially eligible articles. Reference lists of eligible reviews and meta-analyses were searched for additional citations. For gray literature, we searched them through OpenGrey and Google Scholar or directly contacted the author if necessary.

### Eligibility criteria and study selection

Two reviewers independently carried out the study selection from the eligible studies based on the following criteria: (1) full-text systematic reviews and meta-analysis published in the English language; (2) searched at least two electronic databases, such as PubMed, EMBASE, Web of Science, and Cochrane Library; and (3) assessing the relationship between care intervention and poor psychological outcome of ICU patients. After removing the duplicated records from screening the title, two reviewers read the full texts independently and removed studies that did not meet the inclusion criteria. For several kinds of literature focusing on the same interventions or including duplicated primary studies, only the latest reviews or meta-analyses with the largest sample size were considered to be included.

### Data extraction and quality appraisal

Two reviewers (ZY and ZL) separately extracted the data from the included literature. Data extracted included the following: author, publication time, research type, exposures, exposure contrast, study design, population, main psychological outcomes, number of primary studies selected in the reviews, number of participants, specific relative risk estimates (risk ratio [RR], odds ratio [OR], mean difference [MD], standardized mean difference [SMD], standard error of measurement [SEM], as reported by the authors of the meta-analysis) together with the corresponding 95% confidence intervals (95% CIs), heterogeneity, bias, and evidence class (Table 1). Any disagreement was resolved in consensus with the rest of the team.

**Table 1:** Psychological outcomes and evidence class reported in included meta-analyses

Author	Year	Exposure	Exposure contrast	Study design	Population	Outcome	Studies, *n*	Participants, *n*	Type of effect size metric	Effect size (95% CI)	95% CI prediction intervals	*P*	*I*2	Egger’s *P*	Small-study effect	Excess significance bias		Evidence class^1^
																**O/E**	***P***	
Gazzato A	2022	ICU diary intervention	Without diaries	RCTs, cohort, case–control studies	Adults	PTSD	6	854	RR	0.82 (0.63, 1.07)	(0.54, 1.26)	0.147	0	0.009	yes	0/0.37	-	NS
						Anxiety	4	456	RR	0.64 (0.29, 1.40)	(0.03, 15.97)	0.264	66.86	0.399	-	1/0.20	0.187	NS
						Depression	4	456	RR	0.71 (0.49, 1.02)	(0.31, 1.59)	0.066	0	0.300	-	0/0.37	-	NS
Yue W	2021	Music therapy	Standard care	RCTs	Preterm infants and their mothers	Behavioral state	5	437	SMD	− 0.35 (− 1.08, 0.39)	(− 3.15, 2.46)	0.357	92.25	0.059	-	3/1.17	0.087	NS
						Stress level	2	90	SMD	− 17.91 (− 22.02, − 13.80)	-	< 0.001	0	-	-	1/2.00	-	Weak
						Maternal anxiety	2	276	SMD	− 2.08 (− 2.37, − 1.79)	-	< 0.001	0	-	-	2/2.00	1	Weak
Fuke R	2018	Early rehabilitation	No early rehabilitation or standard care	RCTs	Adult patients with critical illness	Hospital Anxiety and Depression Scale (HAS/HADS) score	2	92	OR	0.79 (0.29, 2.14)	-	0.640	-	-	-	0/0.40	-	NS
						QOL scores	2	63	SMD	0.11 (− 0.86, 1.09)	-	0.819	72.21	-	-	0/0.20	-	NS
						SF-36 PF	2	191	SMD	0.78 (0.01, 1.56)	-	0.049	0	-	-	1/0.23	-	Weak
Rosa RG	2019	Post-ICU follow-up	Usual care	RCTs or non-RCTs	Adults	Symptoms of depression	4	147	SMD	− 2.20 (− 4.51, 0.11)	-	0.062	0	-	-	0/0.94	-	NS
						Anxiety symptoms	4	147	SMD	− 1.90 (− 4.44, 0.64)	-	0.143	0	-	-	0/1.00	-	NS
						PTSD short term 0–3 months	4	574	SMD	− 0.45 (− 0.83, − 0.08)	-	0.671	0	-	-	1/0.26	0.258	Weak
						PTSD medium term 3–6 months	4	594	SMD	− 0.30 (− 0.57, − 0.03)	-	0.029	0	-	-	0.23/1.00	0.225	Weak
						PTSD long term > 6 months	3	510	SMD	− 0.16 (− 0.53, 0.21)	-	0.391	62.18	-	-	1/0.42	0.377	NS
						Quality of life (short term 0–3 months)	4	293	SMD	0.65 (0.14, 1.16)	-	0.012	0	-	-	1/0.29	0.289	Weak
						Quality of life (medium term 3–6 months)	2	207	SMD	0.14 (− 0.13, 0.42)	-	0.308	0	-	-	0/0.11	-	NS
Ng SX	2022	Preoperative education	Usual care	RCTs	Adults	Preoperative anxiety	6	657	SMD	− 0.90 (− 1.73, − 0.07)	(− 3.82, 2.02)	0.033	94.75	0.507	-	2/0.53	0.093	Weak
						Postoperative anxiety	6	623	SMD	− 0.49 (− 0.71, − 0.26)	(− 1.08, 0.11)	< 0.001	43.70	0.108	-	5/0.73	< 0.001	Weak
						Depression	3	397	SMD	− 0.21 (− 0.41, − 0.02)	(− 1.50, 1.07)	0.033	0	0.554	-	1/0.32	0.287	Weak
Erbay Dalli Ö	2022	Music interventions	Standard care or noise cancellation	RCTs	Adults	Anxiety	9	638	SMD	− 1.81 (− 3.11, − 0.50)	(− 6.91, 3.30)	0.007	97.48	0.012		9/6.04	0.126	Weak
Brooke J	2012	Information interventions	Standard care	RCTs	Patients and family members	Transfer anxiety	5	629	OR	0.32 (0.09, 1.17)	(0.00, 117.16)	0.085	87.22	0.577	-	2/0.22	0.017	NS
DeForge CE	2022	Communication and psychological support interventions	Usual care	RCTs	Adults	Anxiety 3 months	5	1561	SEM	− 0.47 (− 1.19, 0.25)	(− 2.62, 1.67)	0.199	51.75	0.922	-	2/3.86	-	NS
						Anxiety 6 months	4	1939	SEM	− 0.70 (− 1.19, − 0.22)	(− 2.19, 0.78)	0.004	23.79	0.876	-	1/3.94	-	Weak
						Depression 3 months	5	1552	SEM	− 0.68 (− 1.14, − 0.22)	(− 1.43, 0.07)	0.004	0	0.667	-	1/4.36	-	Weak
						Depression 6 months	5	2127	SEM	− 0.25 (− 1.15, 0.64)	(− 3.32, 2.81)	0.576	76.95	0.906	-	3/0.43	0.006	NS
						Post-traumatic stress 3 months	6	1659	SEM	− 0.25 (− 0.49, − 0.01)	(− 1.03, 0.54)	0.040	79.47	0.716	-	3/2.03	0.413	Weak
						Post-traumatic stress 6 months	5	2126	SEM	− 0.08 (− 0.27, 0.12)	(− 0.76, 0.60)	0.433	77.26	0.979	-	1/0.30	0.262	NS
Bibas L	2019	Surrogate Decision-making Interventions	standard care	RCTs	Adults	Anxiety	5	1365	SMD	− 0.10 (− 0.28, 0.08)	(− 0.68, 0.47)	0.265	69.08	0.727	-	1/0.26	0.235	NS
						Depression	5	1365	SMD	− 0.10 (− 0.28, 0.08)	(− 0.68, 0.47)	0.265	69.08	0.727	-	1/0.26	0.235	NS
						Posttraumatic stress disorder (PTSD)	4	1337	SMD	− 0.04 (− 0.20, 0.12)	(− 0.76, 0.68)	0.633	77.00	0.898	-	2/0.20	0.014	NS

^1^Evidence class criteria—class I (convincing): statistical significance at *P* < 10^−6^, > 1000 cases (or > 20,000 participants for continuous outcomes), the largest component study reported a significant effect (*P* < 0.05); the 95% prediction interval excluded the null, no large heterogeneity (*I*^2^ < 50%), no evidence of small-study effects (*P* > 0.10) and excess significance bias (*P* > 0.10); class II (highly suggestive): significance at *P* < 10^−6^, > 1000 cases (or > 20,000 participants for continuous outcomes), the largest component study reported a significant effect (*P* < 0.05); class III (suggestive): statistical significance at *P* < 10^−3^, > 1000 cases (or > 20,000 participants for continuous outcomes); and class IV (weak): the remaining significant associations at *P* < 0.05

### Data analysis

According to the criteria for classification of the credibility of the evidence used in the previous umbrella reviews [16, 17], we classified the strength of evidence in the following categories: class I (convincing)—statistical significance at *P* < 10^−6^, > 1000 cases (or > 20,000 participants for continuous outcomes), the highest weighted study reported a significant effect (*P* < 0.05); the 95% prediction interval excluded the null, no large heterogeneity (*I*^*2*^ < 50%), no evidence of small-study effects (*P* > 0.10), and excess significance bias (*P* > 0.10); class II (highly suggestive)—significance at *P* < 10^−6^, > 1000 cases (or > 20,000 participants for continuous outcomes), the largest component study reported a significant effect (*P* < 0.05); class III (suggestive)—statistical significance at *P* < 10^−3^, > 1000 cases (or > 20,000 participants for continuous outcomes); and class IV (weak)—the remaining significant associations at *P* < 0.05.

The results were presented based on each intervention and psychological outcomes. For each meta-analysis, we estimated the effect size and its 95% CI using fixed and random effect models [18, 19]. Between-study heterogeneity was estimated by calculating the *I*^*2*^ measure: ≥ 50% values represented high heterogeneity, while > 75% values represented very high heterogeneity [20, 21]. In addition, we used the regression asymptotic test developed by Egger et al. to calculate the evidence of small research effects [22]. We estimated the standard deviation (SD) of the effect size in each meta-analysis to determine if the SD was less than 0.10 in the largest study. Both the small study and excess significance tests were considered significant at *P* < 0.05, which evaluated whether there were too many studies with significant results (i.e., *P* < 0.05) based on the effect power sets at *α* = 0.05 [23]. Statistical analyses were conducted by Stata version 12.1 and *P* values were two-tailed.

The MeaSurement Tool to Assess Systematic Reviews 2 (AMSTAR 2) checklist [24] was used to evaluate the methodological quality of the included systematic reviews and meta-analyses (Table 2). AMSTAR 2 used sixteen items to assess the methodological quality of systematic reviews based on the validity of review design, literature screening, data extraction, and individual study quality assessment. Among these items, AMSTAR 2 designated seven “critical domains” that can critically affect the validity of a review (e.g., items 2, 4, 7, 9, 11, 13, and 15). Meta-analyses were considered as “high quality” if they met all “critical domains” with other items satisfied ≥ 8, and other meta-analyses that met all “critical domains” were regarded as “medium quality.” Besides, meta-analyses with one unsatisfied critical domain were assigned to “low quality,” and meta-analyses with more than one unsatisfied critical domain were considered as “critically low quality” [24].

**Table 2:** Methodological quality assessment of included studies using AMSTAR 2

Study	Item 1	Item 2	Item 3	Item 4	Item 5	Item 6	Item 7	Item 8	Item 9	Item 10	Item 11	Item 12	Item 13	Item 14	Item 15	Item 16	Overall rating
Gazzato et al. [3]	1	1	1	1	1	1	1	1	0	1	1	1	1	1	0	1	Medium
Yue et al. [25]	1	1	1	1	1	1	1	1	0	1	1	1	1	1	0	1	Medium
Fuke et al. [26]	1	1	1	1	0	0	1	1	1	1	1	1	1	1	1	0	High
Rosa et al.[27]	1	1	1	1	0	0	1	1	1	1	1	1	1	0	1	1	High
Ng et al. [28]	1	1	1	1	1	1	1	1	0	1	1	1	0	1	1	1	Medium
Erbay Dalli et al. [29]	1	1	1	1	1	1	1	1	0	1	1	1	1	1	0	1	Medium
Brooke et al. [30]	1	1	1	1	0	0	1	1	1	0	1	1	1	1	1	1	High
DeForge et al. [31]	1	1	1	1	1	1	1	1	0	1	1	1	1	1	0	1	Medium
Bibas et al. [32]	1	1	1	1	1	1	1	1	1	0	1	1	1	1	1	0	High

AMSTAR 2 (128) used sixteen items to assess methodological quality of systematic reviews on the basis of the validity of review design, literature screening, data extraction, and individual study quality assessment. Among these items, AMSTAR 2 designated seven “critical domains” that can critically affect the validity of a review (e.g., items 2, 4, 7, 9, 11, 13, and 15). Meta-analyses were considered as high quality if they met all “critical domains” with other items satisfied ≥ 8, and other meta-analyses that met all “critical domains” were considered as medium quality. Besides, meta-analyses with one unsatisfied critical domain were assigned to low quality, and meta-analyses with more than one unsatisfied critical domain were considered as critically low quality. The items are as follows:

Item 1: Did the research questions and inclusion criteria for the review include the components of PICO?

Item 2: Did the report of the review contain an explicit statement that the review methods were established prior to conduct of the review and did the report justify any significant deviations from the protocol?

Item 3: Did the review authors explain their selection of the study designs for inclusion in the review?

Item 4: Did the review authors use a comprehensive literature search strategy?

Item 5: Did the review authors perform study selection in duplicate?

Item 6: Did the review authors perform data extraction in duplicate?

Item 7: Did the review authors provide a list of excluded studies and justify the exclusions?

Item 8: Did the review authors describe the included studies in adequate detail?

Item 9: Did the review authors use a satisfactory technique for assessing the risk of bias (RoB) in individual studies that were included in the review?

Item 10: Did the review authors report on the sources of funding for the studies included in the review?

Item 11: If meta-analysis was justified, did the review authors use appropriate methods for statistical combination of results?

Item 12: If meta-analysis was performed, did the review authors assess the potential impact of RoB in individual studies on the results of the meta-analysis or other evidence synthesis?

Item 13: Did the review authors account for RoB in individual studies when interpreting/discussing the results of the review?

Item 14: Did the review authors provide a satisfactory explanation for, and discussion of, any heterogeneity observed in the results of the review?

Item 15: If they performed quantitative synthesis, did the review authors carry out an adequate investigation of publication bias (small-study bias) and discuss its likely impact on the results of the review?

Item 16: Did the review authors report any potential sources of conflict of interest, including any funding they received for conducting the review?

## Results

### Results of the search process

**Fig. 1:**
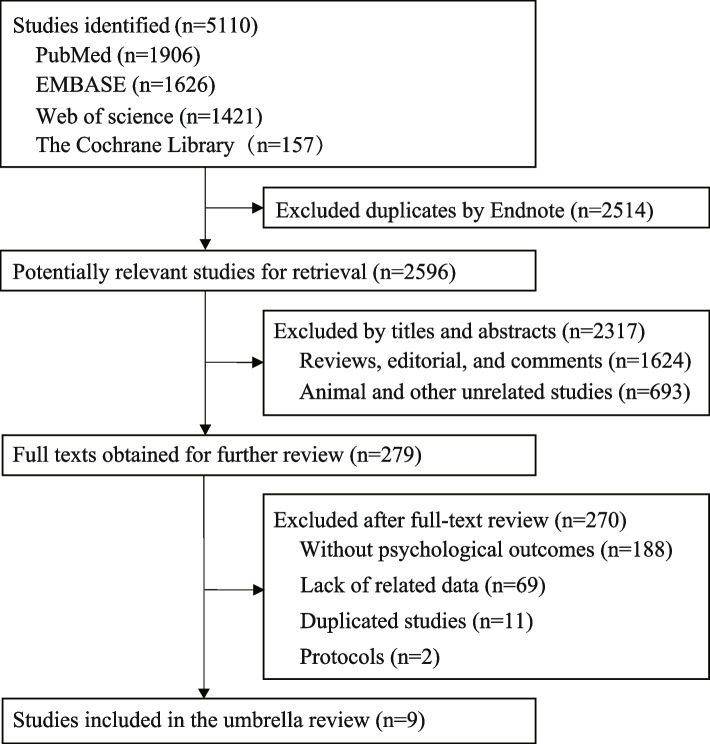
Flow chart

A total of 5110 articles were retrieved from four databases. Of them, 2514 duplicates were removed, and 2317 articles were excluded after screening the titles and abstracts (1624 traditional reviews, reviews, and academic reports; 693 animal and other unrelated studies). Assessment of full-text screening resulted in the exclusion of 270 articles, including 188 articles excluded due to missing psychiatric outcomes, 69 lacking important relevant data, 11 studies investigating duplicate interventions [33–43], and 2 publications being a protocol [44, 45]. Subsequently, 9 articles were included in the umbrella review (Table S2). The flow chart of the study selection is depicted in Fig. 1.

### Description of included systematic reviews

The publication dates of the 9 reviews ranged from 2012 to 2022. The publication dates of the reviews ranged from 1987 to 2020. There were 127 trials. The participant numbers were up to 55,000. The general characteristics of the systematic reviews are presented in Table 1. The majority of the reviews were graded as “medium” to “high quality” based on the AMSTAR 2 score. However, the evidence class of most reviews included was weak. A summary of the quality appraisals of the reviews and the AMSTAR 2 scores are presented in Table 2.

### Review findings

The 9 systematic reviews evaluated 8 interventions: music therapy, early rehabilitation, post-ICU follow-up, ICU diary, information intervention, preoperative education, communication and psychological support, and surrogate decision-making.

### Music therapy

Music therapy refers to the use of music-based interventions within a therapeutic relationship to accomplish individualized goals [25]. Two reviews previously examined the effectiveness of music on intensive care patients [25, 29]. Of them, Yue et al. [25] reviewed the impact of music therapy on neonates in ICU and found that music therapy had a significant influence on preterm infants’ heart rate and respiratory rate and exerted a beneficial effect on oral feeding volume. In addition, music therapy was also found to play a role in reducing maternal anxiety [25]. However, due to the heterogeneity across studies in some outcomes, further studies with larger sample sizes and more stringent designs are needed [25]. Another review was to explore the impact of music therapy on the physiological and psychological stress response of patients in ICU [29]. Music was found to significantly reduce anxiety scores with an SMD of − 1.97 (95% CI =  − 3.66 to − 0.28; *n* = 6) compared to standard care (*P* = 0.02), but there was no significant change in anxiety scores in comparison with the noise cancellation group (*P* = 0.14) [29]. It was found that multiple music sessions reduced the anxiety level better than a single music session [29].

### Intensive care unit diaries

Intensive care unit diaries include the daily events of patients and may allow patients to reconstruct their experience [3]. A review conducted by Gazzato et al. included 7 RCTs and examined the effect of ICU diaries on PTSD, anxiety, and depression [3]. They found patients who received a diary during the ICU admission had a reduced rate of PTSD (78/432 [18%] *vs*. 106/422 [25%]; *RR* = 0.73 [95% CI, 0.57 to 0.94; *n* = 6]; *P* = 0.02; *I*^*2*^ = 0%; trial sequential analysis-adjusted CI, 0.55 to 0.97), compared to patients who did not receive a diary [3].

### Early rehabilitation

One review included six randomized controlled trials (RCTs) examining the effectiveness of early rehabilitation for the prevention of PICS, which was characterized by an impaired physical, cognitive, or mental health status, among survivors of critical illness [9, 26]. This review found that early rehabilitation significantly improved short-term physical-related outcomes, as indicated by an increased Medical Research Council scale score (SMD = 0.38 [95% CI, 0.10 to 0.66; *n* = 3], *P* = 0.009, qualify of evidence [QoE]: low) and a decreased incidence of ICU-acquired weakness (OR = 0.42 [95% CI, 0.22 to 0.82; *n* = 2], *P* = 0.01, QoE: low), compared with standard care or no early rehabilitation [26]. However, these two groups did not differ in terms of cognitive-related delirium-free days (SMD =  − 0.02 [95% CI =  − 0.23 to 0.20; *n* = 2], QoE: low) and the mental health-related hospital anxiety and depression scale score (OR = 0.79 [95% CI = 0.29 to 2.12; *n* = 2], QoE: low) [26].

### Post-ICU follow-up

Post-ICU follow-up aimed to minimize post-ICU burden for patients, families, and the health care system. Based on 26 studies, one review synthesized data on subject outcomes associated with post-ICU follow-up [27]. In an RCT, post-ICU follow-up models focusing on physical therapy were associated with fewer depression symptoms (MD =  − 1.21 [95% CI, 2.31 to − 0.11; *n* = 4]; *I*^*2*^ = 0%) and better mental health-related quality of life scores (SMD = 0.26 [95% CI, 0.02 to 0.51; *n* = 4]; *I*^*2*^ = 6%) in the short term [27]. Post-ICU follow-up models focusing on psychological or medical management interventions were associated with fewer PTSD symptoms (SMD =  − 0.21 [95% CI, − 0.37 to − 0.05; *n* = 4]; *I*^*2*^ = 0%) in the medium term [27].

### Information intervention

Information intervention means providing patients and their families with disease-related information or future medical environmental information. Brooke et al. [30] undertook a comprehensive systematic review on the efficacy of information intervention, which might reduce anxiety in patients and family members during the time when patients were transferred from a critical care setting to a general ward. They found family members’ transfer anxiety was significantly reduced in the intervention group of information provision (OR = 1.70 [95% CI, 1.15 to 2.52; *n* = 4]; *P* = 0.01), related to those who received standard care (OR = 0.42 [95% CI, 0.28 to 0.63; *n* = 3]; *P* < 0.001) [30].

### Communication and enhancing psychological support

One review evaluated the efficacy of interventions to improve symptoms for ICU surrogates at the highest risk of developing psychological distress [31]. This study showed that communication and enhancing psychological support from the ICU could alleviate the anxiety, depression, and posttraumatic stress of ICU surrogates facing end-of-life decisions to some extent. Significant improvement was seen at 3 months (depression: MD =  − 0.68 [95% CI, − 1.14 to − 0.22; *n* = 5], moderate certainty; posttraumatic stress: SMD =  − 0.25 [95% CI, − 0.49 to − 0.01; *n* = 6], very low certainty) and 6 months (anxiety: MD =  − 0.70 [95% CI, − 1.18 to − 0.22; *n* = 4], moderate certainty) [31].

### Preoperative education interventions

Preoperative education was defined as the basic element to enhance the postoperative recovery path, which aims to provide patients with psychological support information, set health expectations, and promote rehabilitation [46]. Preoperative education was known to positively alter people’s perceptions, and emotions, and mitigate surgical distress [47]. One review examined this intervention’s effectiveness in improving perioperative outcomes among patients undergoing cardiac surgery [28]. They found that preoperative education exerted greatly significant effects on reducing post-intervention preoperative anxiety (*P* = 0.02) and improving knowledge (*P* < 0.001), but they also found small significant effect sizes on lowering postoperative anxiety (*P* < 0.001), depression (*P* = 0.03), and enhancing satisfaction (*P* = 0.04) [28].

### Surrogate decision-making intervention

Surrogate decision-making (SDM) intervention refers to making important decisions on behalf of critically ill patients during the time when ICU patients’ lose decision-making ability. One review conducted by Bibas et al. [32] determined the association of SDM interventions with patient- and family-centered outcomes and resource use. This review screened 3735 studies, comprising a total of 10,453 patients from 13 RCTs. It includes 4 types of interventions: care professional-led, ethics consultation, palliative care consultation, and media. There was no consistent difference in SDM-related outcomes, including satisfaction with care or perceived quality of care (*n* = 6) and incident psychological comorbidities (depression: ratio of means =  − 0.11 [95% CI, − 0.29 to 0.08; *n* = 5], *P* = 0.26; anxiety: ratio of means =  − 0.08 [95% CI, − 0.25 to 0.08; *n* = 5], *P* = 0.31; or PTSD: ratio of means =  − 0.04 [95% CI, − 0.21 to 0.13; *n* = 4], *P* = 0.65) [32].

## Discussion

### Summary of main findings

This umbrella review provided an overview of reported findings on the impact of care intervention on the psychological outcome of ICU patients. Overall, eight classes of interventions have been identified for the association, including ICU diary intervention, music therapy/interventions, early rehabilitation, post-ICU follow-up, preoperative education, information interventions, communication and psychological support interventions, and surrogate decision-making interventions. Most of the selected reviews were of “medium” to “high” methodological quality. After assessing the strength direction, we found that each type of care intervention can improve the psychological outcomes of ICU patients, but this finding was supported by evidence with low epidemiological credibility, as expressed by small sample size and large heterogeneity. Among these studies, one study is not suggestive due to the small-study effect.

On the basis of the results of this umbrella review, ICU diary intervention could reduce the incidence of PTSD symptoms in ICU patients [3]. Communication and psychological support intervention in ICU have made minor but significant improvements in improving the psychological symptoms of relatives who took the place of ICU patients to make terminal decisions [31]. However, our assessment failed to show an overall level of evidence class of this study, and its small-study effect and the medium methodological quality suggested that this evidence should be treated with caution.

Compared with standard daily care, music therapy could not only improve the infant’s eating behavior in ICU premature infants but also alleviate their mother’s anxiety [25]. This may be explained by the influence of music on the periphery and autonomic nervous system of premature infants [48]. Prior research showed that music could help coordinate the function of the cerebral cortex, thereby improving the physiological function of the brain [49]. Ranger et al. also found that music intervention could reduce mothers’ anxiety [50]. Additionally, music intervention involving multiple sessions could be used as a care intervention to control the anxiety level of ICU patients [29]; this might be ascribed to the fact that sound signals might reduce noise and improve harmony more than verbal signals [51]. However, the included meta-analysis did not find a statistically significant effect of music therapy on improving the oxygen saturation or behavioral status of premature infants [25]. This may be related to the kangaroo care effect, which was reported by two experiments that combined kangaroo care and music therapy as intervention measures [52]. The effect of this combined intervention might be partly explained by that thermal stimulation could significantly weaken or mask other stimuli, including various types of music. However, our study suggested that there was probably genuine heterogeneity (92.3%) in the association of music therapy with behavior stats in infants. This high heterogeneity might be not only related to the potential bias in the original studies but also related to the differences across studies included in this meta-analysis. Furthermore, being graded as medium methodological quality, these associations were both supported by a weak level of evidence; thus, more studies are needed to further document the effect of music therapy on mental health in ICU patients.

Providing information to the ICU patients and their caregivers about the future ward environment could significantly reduce their anxiety when transferring patients from the intensive care environment [30]. Despite this study being graded as having high methodological quality, the level of research evidence was weak due to the small number of subjects included in the study. When patients and their families cannot understand the information provided by ICU physicians or get less information, their uncertainty would increase, which would further cause them anxiety. Research showed that uncertainty accounted for 30.2% of the factors that cause family members’ transfer anxiety, and providing information was a key factor in reducing uncertainty and anxiety [52, 53].

With the medium methodological quality, preoperative education was found to be helpful in improving the prognosis of patients undergoing cardiac surgery [28] because it may enhance patients’ knowledge; besides, meeting their information needs could also mitigate their distress [54]. However, our umbrella-shaped review shows that information intervention cannot effectively reduce patients’ transfer anxiety, which may be related to our use of quantitative rather than qualitative methods to investigate transfer anxiety. This indicates that more high-quality systematically conducted studies are needed to better understand the associations between providing information and adverse outcomes in ICU patients.

With high methodological quality, early rehabilitation was found to be effective in the improvement of short-term physical outcomes in critically ill patients [49]. Kayambu et al. [55] consistently found a similar conclusion that physiotherapy in the ICU could improve muscle strength, body function, quality of life, and days without convulsion and reduce ICU hospitalization time and hospitalization time. However, a study has inversely shown that acute rehabilitation may cause great physical pressure and fatigue to ICU patients, thus increasing mortality in the ICU [56]. Nonetheless, the results of the study by Fuke et al. [26] showed that early rehabilitation did not significantly improve the cognitive and mental state of patients. The possible reason for this discrepancy might be that the review shed light on studies that implemented multiple “early rehabilitation” programs, and further analysis of large-scale trials was still needed in the later stage, with detailed records and grouping analysis made on the time, type, and intensity of early rehabilitation. Therefore, whether early rehabilitation contributed to the recovery of intensive care syndrome still needs further research.

Post-ICU follow-up focusing on physical therapy could improve depressive symptoms and mental health-related quality of life in the short term, while post-ICU follow-up focusing on psychological or medical management intervention can improve PTSD symptoms in the medium term [27]. However, the evidence supporting these findings was mainly of weak evidence class, though this association originated from a review with high methodological quality. It may be attributable to the fact that the individual risk factors of ICU patients were not considered when the subjects were included in the study. The high heterogeneity of the ICU population, therefore, may offset the potential benefits of nursing intervention on the adverse psychological outcomes of ICU patients and their caregivers. Optimizing the inclusion criteria for follow-up patients after ICU and conducting specific treatments for subjects, who may benefit from specific rehabilitation strategies, may lead to more accurate intervention effects. Given that these results are mainly from non-randomized studies, which may also be the reason for the low evidence class, carefully designed randomized trials were still needed to further verify the impact of follow-up in ICU on the psychological outcomes of patients and their families.

Alternative SDM itself could only shorten the stay time of dead patients in the ICU, but it would not affect the overall mortality of ICU patients [32]. It might be ascribed that surrogate decision-making is a complex task in the ICU environment, and the prognosis judgment of ICU doctors may be wrong. Thus, the surrogate decision-making interventions may cause patients to lose life support prematurely; otherwise, this part of patients may survive for a longer time [57]. In addition, personal characteristics related to SDM, such as coping strategies and competitive responsibilities, may also affect the effect of this intervention. People who have faced similar situations before tend to do the job better than those who have served as SDM for the first time [58, 59]. With the high methodological quality, the assessments of these meta-analyses related to alternative decision-making intervention had high between-study heterogeneity, as well as their null evidence class, suggesting that there is no clear scientific evidence to support the link between alternative decision-making intervention and adverse outcomes in ICU patients.

### Limitation of the overview

This umbrella review has several limitations. Firstly, this review was conducted based on the results of the published systematic review and meta-analysis; thus, it is inevitable to suffer from missing data from the original literature and their relevant literature. However, our result was not greatly affected because the evaluation of repeated meta-analysis led to similar results. Secondly, the statistical method we used to test the existence of bias could only indicate the existence of bias, but could not prove its exact source. Finally, our estimation method was relatively conservative, but the detection showed no bias, which did not rule out the possibility of its existence.

## Conclusion

The evidence reviewed here indicates that ICU diary intervention, music therapy, early rehabilitation, post-ICU follow-up, preoperative education, information intervention, communication and psychological support intervention, and alternative decision-making may be beneficial to ICU patients and caregivers. However, further high-quality population experiments are still needed to further demonstrate these associations because the evidence supporting our finding was mainly of null or weak evidence class.

### Supplementary Information

**Additional file 1: Table S1.** Search strategy for the identification of systematic reviews and meta-analyses for PubMed. **Table S2.** The details of five reviews being excluded due to the duplication.

## Data Availability

All data generated or analyzed during this study are included in this published article (and its supplementary information files).

## Abbreviations

95% CIs: 95% Confidence intervals
AMSTAR-2: Assessment of Multiple Systematic Reviews 2
ICU: Intensive care unit
MD: Mean difference
OR: Odds ratio
PICS: Post-intensive care syndrome
PRISMA: Preferred Reporting Items for Systematic Reviews and Meta-Analyses
PTSD: Post-traumatic stress disorder
RR: Risk ratio
SMD: Standardized mean difference
