# Supplementary material for: Care intervention on psychological outcomes among patients admitted to intensive care unit: an umbrella review of systematic reviews and meta-analyses
Source: Syst Rev. 2023 Dec 14;12:237. doi: 10.1186/s13643-023-02372-5 (PMC10720116; doi:10.1186/s13643-023-02372-5)
Supplement: Supplementary file 1 — Additional file 1: Table S1. Search strategy for the identification of systematic reviews and meta-analyses for PubMed. Table S2. The details of five reviews being excluded due to the duplication. [file 13643_2023_2372_MOESM1_ESM.docx]

**Table S1: Search strategy for the identification of systematic reviews and meta-analyses for PubMed**

| **Step** | **Searching key words** | **Number of potential studies** |
| --- | --- | --- |
| #1 | "Mental Disorders"[Mesh] OR psychiatric OR psychological* OR mental disorder* OR dysfunction* OR psychogenetic OR stuttering OR autis* OR Asperger OR ASD OR "attention deficit disorder with hyperactivity" OR "attention deficit hyperactivity disorder" OR "disturbance of activity and attention" OR ADHD OR ADD OR pica OR encopresis OR enuresis OR "selective mutism" OR "acquired aphasia with epilepsy" OR "Rett´s syndrome" OR "selective mutism" OR cluttering OR delirium OR Alzheimer OR dementia OR "amnesic syndrome" OR hallucinosis OR "postencephalitic syndrome" OR "postconcussional syndrome" OR abuse OR dependence OR intoxication OR withdrawal OR "acute intoxication" OR "harmful use" OR schizophrenia OR SCZ OR psycho* OR psychotic OR depress* OR manic OR mania OR hypomania OR bipolar OR cyclothymi* OR dysthymi* OR agoraphobia OR phobi* OR PTSD OR anxiety OR "predominantly obsessional thoughts or ruminations" OR "obsessive compulsive disorder" OR OCD OR obsession* OR compulsion* OR "predominantly compulsive acts" OR "acute stress reaction" OR "reaction* to severe stress" OR dissociate* OR hypochondriasis OR neurasthenia OR "depersonalization-derealization syndrome" OR "premature ejaculation" OR vaginismus OR dyspareunia OR paraphilia OR exhibitionism OR fetishism OR pedophilia OR "sexual masochism" OR "sexual sadism" OR voyeurism OR "sexual aversion" OR "sexual desire" OR "failure of genital response" OR "excessive sexual drive" OR "inhibited male orgasm" OR transsexualism OR transvestism OR paedophilia OR sadomasochism OR "egodystonic sexual orientation" OR "inhibited female orgasm" OR frotteurism OR anorexia OR overeating OR bulimia OR dyssomnia OR insomnia OR hypersomnia OR narcolepsy OR parasomnia OR "restless legs syndrome" OR "sleep walking" OR "sleep terror" OR "sleep disorder*" OR "abuse of non-dependence-producing substances" OR "behavioural syndrome" OR "behavioral syndrome" OR kleptomania OR pyromania OR "pathological gambling" OR trichotillomania OR retardation OR personality disorder* | 7,420,620 |
| #2 | ICU OR “intensive care unit” | 252,437 |
| #3 | "Systematic Review"[Publication Type] OR "Systematic Reviews as Topic"[Mesh] OR "Meta-Analysis as Topic"[Mesh] OR "Meta-Analysis"[Publication Type] OR “systematic review" OR “systematic literature review" OR “meta-analysis" OR “meta-analyses" OR “meta analysis" OR “meta analyses" | 426,655 |
| #4 | #1 AND #2 AND 3 | 1906 |

For all databases, the original search date was October 1, 2020, and was updated on January 3, 2023.

**Table S2: The details of five reviews being excluded due to the duplication.**

| **Author** | **Year** | **Title** | **Exposure** | **Outcome** |
| --- | --- | --- | --- | --- |
| Bieleninik Ł | 2016 | Music Therapy for Preterm Infants and Their Parents: A Meta-analysis | Music Therapy | Infant respiratory rate and maternal anxiety |
| Nydahl P | 2018 | Diaries for intensive care unit patients reduce the risk for psychological sequelae: Systematic literature review and meta-analysis | Intensive care unit diary | Posttraumatic stress disorder in patients or relatives |
| Sun XH | 2021 | Effect of intensive care unit diary on incidence of posttraumatic stress disorder, anxiety, and depression of adult intensive care unit survivors: A systematic review and meta-analysis | Intensive care unit diary | Posttraumatic stress disorder, anxiety, and depression in patients |
| Kang JY | 2018 | Effect of nonpharmacological interventions for the prevention of delirium in the intensive care unit: A systematic review and meta-analysis | Nonpharmacological interventions | Delirium |
| Mohan A | 2021 | Music therapy for preterm neonates in the neonatal intensive care unit: An overview of systematic reviews | Music therapy | Physiological parameters in preterm infants |
| BB Barreto | 2019 | The impact of intensive care unit diaries on patients' and relatives' outcomes: a systematic review and meta-analysis | Intensive care unit diary | Psychiatric disorders in patients and relatives |
